# How high is the quality of the videos about children's elbow fractures on Youtube?

**DOI:** 10.1186/s13018-023-03648-1

**Published:** 2023-03-03

**Authors:** Aybars Kıvrak, İbrahim Ulusoy

**Affiliations:** 1 Department of Orthopedics and Traumatology, Adana Avrupa Hospital, Adana, Türkiye; 2 Department of Orthopedics and Traumatology , Selahaddin Eyyubi State Hospital, Diyarbakır, Türkiye

**Keywords:** Elbow fractures, Youtube, Child, Video

## Abstract

**Objective:**

Pediatric elbow fractures are children's most common fractures. People use the internet to get information about their illnesses and also to search for treatment options. Videos uploaded to Youtube do not go through the review process. The aim of our study is to determine the quality of videos related to child elbow fractures on Youtube.

**Material method:**

The study was carried out with the data obtained from the video-sharing site www.youtube.com on 01.12.2022. Pediatric elbow fractures are written in the search engine section. Information about the number of views of the videos, upload date, view rate (number of views per day), number of comments, number of likes and dislikes, duration, whether they contain animation and the publishing source were evaluated. The videos are divided into 5 groups according to their sources: medical society/non-profit organization, physician, healthrelated website, university/academic, and patient/independent user/other. The quality of the videos was evaluated using the Global Quality Scale (GQS). All videos have been evaluated by 2 researchers.

**Results:**

In the study, 50 videos were included. In the statistical evaluation, no significant correlation was found between the modified discern score and GQS found by both researchers, and the number of views, the rate of views, comments, likes and dislikes, video duration and VPI. In addition, when the GQS and modified discern scores were compared according to the source of the video, it was found that the patient/independent user/other group scores were lower numerically, but there was no statistically significant difference in comparison.

**Conclusion:**

Most of the videos about child elbow fractures have been uploaded by healthcare professionals. Therefore, we concluded that the videos are quite informative in terms of accurate information and quality content.

## Introduction

Pediatric elbow fractures are children's most common fractures [1]. While some of these fractures are followed by surgical intervention, some of them are followed conservatively. Especially in the families of patients who are offered surgical intervention, there may be question marks in their minds. Families begin to do research to eliminate these question marks.

As a result of the internet access in every home with the developing technology, people actively use the internet to access information. Youtube is one of the video-sharing platforms used to access information. This platform, which is used by 95% of internet users, has more than 30 million daily active users [2]. People use the internet to get information about their illnesses and also to search for treatment options. Its popularity has been increasing recently due to the videos shared on Youtube. Videos uploaded to Youtube do not go through the review process. Most videos do not provide information about the author or source. The videos on this video-sharing platform, where everyone can easily upload and watch content, raise doubts about the quality and accuracy of the information. The educational aspect of YouTube videos for some orthopedic diseases and their treatment has been evaluated in many studies. Another point that is as important as easy access to information is to reach the right information. The aim of our study is to determine the quality of videos related to child elbow fractures on Youtube.

## Material method

The study was carried out with the data obtained from the video-sharing site www.youtube.com on 01.12.2022. In order not to affect the results, the search history of the internet browser was deleted and a search was made. Standard settings are used when searching. There are studies in which all videos are evaluated, as well as studies using a specific sample [3, 4]. Pediatric elbow fractures are written in the search engine section. Only English videos are included in the study. Videos that do not contain sound, that are not relevant to the subject, and that contain advertisements were excluded from the study. Considering the exclusion criteria, as in previous studies, the first 50 videos were included in our study [2].

Information about the number of views of the videos, upload date, view rate (number of views per day), number of comments, number of likes and dislikes, duration, whether they contain animation and the publishing source were evaluated. In addition, the popularity of the videos was included in the evaluation using the video power index (VPI) [(number of likes/number of likes + number of dislikes) × 100] [5].

The videos are divided into 5 groups according to their sources: medical society/non-profit organization, physician, healthrelated website, university/academic, and patient/independent user/other. The quality of the videos was evaluated using the Global Quality Scale (GQS). Scoring in the GQS ranges from 1 to 5, with 5 representing high quality and 1 representing low quality. The details of the Global Quality Scale are given in Table 1 [6].

**Table 1:** GQS (Global Quality Scale)

Score	Description
1	Low quality, poor flow of the site, missing most of the information, not useful for patients at all
2	Generally poor quality and poor site flow. Some information is available but many important issues are missing, very limited use for patients
3	Generally poor quality and poor site flow. Some information is available but many important issues are missing, very limited use for patients
4	The quality is high and the streaming is good overall. It contains most of the relevant information, but some topics are missing, useful for patients
5	Its quality and broadcast flow are at a high level, very useful for patients. It gives complete and clear information

The reliability and integrity of the information in the videos were evaluated using the modified discern scale [7]. Scoring ranges from 0 to 5. A high score indicates high reliability. The details of the modified discern scale are given in Table 2.

**Table 2:** Modified DISCERN Scale (Yes: 1, No: 0 points for each question)

Score	Questions
1/0	Is the video clear, short and understandable?
1/0	Is it obtained from valid sources?
1/0	Is the information presented balanced and unbiased?
1/0	Are additional sources of information for the patient/audience specified?
1/0	Does the video consider controversial or ambiguous topics?

All videos have been evaluated by 2 researchers. The videos were watched once and the evaluation was made by the researchers according to the modified discern score and GQS criteria.

Reliability analysis measures the consistency of responses to a questionnaire prepared according to a predetermined type of scale. Here, what is meant by consistency is the consistency of the answers given to questions that contain only ordinal scale answers. Cronbach α was used for the determination of inter-rater consistency.

Ethics committee approval is not required for this study. Data from a public website were used, and no human participants/animals were used. Ethics committee approval was not required for similar studies [8–10]

Data obtained in the study were analyzed statistically using the Statistical Package for the Social Sciences version 29.0.0.0 (241) software (SPSS Inc., Chicago, IL, USA). Descriptive data were stated as number, percentage, and median (Interquartile Range-IR) values. Conformity of the data to normal distribution was checked using the Shapiro–Wilk test. Kruskal–Wallis, Spearman and Mann–Whitney U tests were used. The agreement between the two physicians was assessed using the Cronbach α. A value of *p* < 0.05 was accepted as statistically significant. Acceptable α value was determined as above 0.7 [11].

## Results

In the study, 50 videos were included. The average number of views of the videos is 36507, and the average duration is 1192 s. General features of the videos are given in Table 3.

**Table 3:** General features of the videos

Video features	Median (IR*)
View Count	13,842 (29,479)
Duration (seconds)	662.5 (818.75)
Total Likes	183 (335.75)
Total Dislikes	4.5 (9.25)
Number of Comments	6 (15.25)
View Rate	11.57 (18.56)
VPI	97.59 (3.47)

*IR Interquartile Range

In the study, 9 of the videos contain animation, 22 of them are real images, and 19 of them are mixed with animation and real image. When the sources of the videos were evaluated, 24 videos were shared by physician, 10 videos by health-related website, 8 videos by medical society/non-profit organization, 5 videos by academic/university, and 3 videos by patient/independent user.

The consistency of the GQS and modified discern scores found by the researchers was determined by Cronbach α (Table 4). The results found by the researchers were consistent according to Cronbach α. In addition, since the results found by the researchers gave similar results in the statistical tests performed separately, there was no need for consensus in the results (Table 5).

**Table 4:** Interobserver reliability of the modified discern and GQS

	Median (IR*)	Cronbach’s alpha
Modified Discern-1	3 (1)	0.934
Modified Discern-2	3 (1)	
GQS-1	3 (1)	0.917
GQS-2	3 (1)	

*IR Interquartile Range

**Table 5:** Average GQS and modified discern scores by video creator

	Number (percent)	GQS-1	GQS-2	Modified Discern-1	Modified Discern-2
Medical society/non-profit organization	8 (16%)	3.25 ± 0.46	3.25 ± 0.7	3.5 ± 0.53	3.25 ± 0.46
Physician	24 (48%)	3.2 ± 0.83	3.29 ± 1.04	3.25 ± 0.94	3.33 ± 0.91
Healthrelated website	10 (20%)	3.3 ± 0.67	3.4 ± 0.69	3.6 ± 0.51	3.5 ± 0.52
University/academic	5 (10%)	3.8 ± 0.83	3.6 ± 0.89	3.8 ± 1.09	3.8 ± 1.09
Patient/independent user/other	3 (6%)	2 ± 0	2.33 ± 0.57	2 ± 0	2 ± 0

In the statistical evaluation, no significant correlation was found between the modified discern score and GQS found by both researchers, and the number of views, the rate of views, comments, likes and dislikes, video duration and VPI (*p* > 0.05). In the evaluation made according to whether the videos contain animation or not, no statistical difference was found between modified discern and GQS scores (*p* > 0.05). In addition, when the GQS and modified discern scores were compared according to the source of the video, it was found that the patient/independent user/other group scores were lower numerically, but there was no statistically significant difference in comparison (*p* > 0.05).

## Discussion

One of the tools that people frequently seek answers to their questions is the internet. Although it is possible to easily access almost all kinds of information on the Internet, it can also mediate the spread of confusing and false information [12].

In our study, videos related to pediatric elbow fractures on Youtube, a video-sharing site frequently used by internet users, were evaluated and it was concluded that the quality and reliability of the video vary according to the source of sharing, and the quality and reliability of the videos shared by independent users or users outside the field of health are low.

Scales such as modified Discern, Journal Of The American Medical Association (JAMA) benchmark criteria, and GQS have been frequently used in similar studies. Modified Discern score was used to measure reliability, GQS was used to measure the quality of videos [2, 3, 5, 6], and JAMA benchmark criteria are used to measure the reliability, relevance and usefulness of websites. The review includes authorship, source, date, last update, and whether there are relevant disclosures (sponsorship, conflicts of interest, profit organization partnership). This scoring was not used in our study, as it was thought that not all of these criteria could be answered or this information could not be accessed during the review of the videos on Youtube.

When we look at the studies in the literature, it is seen that the number of patients or non-health professionals uploading videos with medical content to the internet is at a level that cannot be underestimated [13]. In our study, we concluded that the shares of individuals who are not health professionals remained at the level of 3%. In addition, when the quality of the videos is considered, it is seen that the GQS and Discern scores are lower in the patient/independent user/other group. It is seen that it is the highest in the University/academic group. If we talk about the subject of our study, it can be concluded that it is easier for video viewers to reach high-quality content, considering that the shares of non-health professionals remain at the level of 3%.

In the literature, there are studies that reach different conclusions about the number of likes, dislikes and views according to the quality of the content. It has been concluded that videos with high-quality content are more popular in some studies, while videos with low-quality content are more popular in some studies [14–16]. In our study, we did not find a significant correlation between the modified discern score and GQS, and the number of views, the rate of views, comments, likes and dislikes, video duration and VPI. There may be many reasons for the lack of consensus in the literature. The low frequency of encountering the medical content searched for in the community may only attract healthcare professionals to this medical content. As a result, the number of likes and views of videos with high-quality content may increase. In addition, medical videos of common diseases in the society can be watched relatively more by non-health professionals. This can produce results independent of video content quality.

There are some limitations of our study. Although it is not unique to our study, there are common limitations of Youtube video reviews. Since Youtube is a dynamic social media sharing area, new videos are constantly being added, and the number of comments, views, likes and dislikes is constantly changing. In addition, although we delete the history in the internet browser, it can be affected by past searches. Although our study may be influenced by the geography of the search, it is limited to only English language videos.

## Conclusion

Most of the videos about child elbow fractures have been uploaded by healthcare professionals. Therefore, we concluded that the videos are quite informative in terms of accurate information and quality content. Channels that are not healthcare professionals or are suspected, videos uploaded by individuals should not be used to obtain information about child elbow fractures.

## Data Availability

The datasets used and analyzed during the current study are available from the corresponding author on reasonable request.
