# Correction: How high is the quality of the videos about children’s elbow fractures on Youtube?

**DOI:** 10.1186/s13018-023-03721-9

**Published:** 2023-03-23

**Authors:** Aybars Kıvrak, İbrahim Ulusoy

**Affiliations:** 1 Department of Orthopedics and Traumatology, Adana Avrupa Hospital, Adana, Türkiye; 2 Department of Orthopedics and Traumatology, Selahaddin Eyyubi State Hospital, Diyarbakır, Türkiye

**Correction: Journal of Orthopaedic Surgery and Research (2023) 18:166** **https://doi.org/10.1186/s13018-023-03648-1**

Following publication of the original article [1], the author reported that the affiliations of the authors are incorrect. The correct version of affiliations is provided in this correction.

The original article has been corrected.
